# Impact of Climate Variability on Foodborne Diarrheal Disease: Systematic Review and Meta-Analysis

**DOI:** 10.3389/phrs.2025.1607859

**Published:** 2025-02-19

**Authors:** Tesfaye Gobena, Dechasa Adare Mengistu

**Affiliations:** School of Environmental Health, College of Health and Medical Science, Haramaya University, Harar, Ethiopia

**Keywords:** foodborne diarrheal disease, diarrheal disease, food safety, climate variability, climatic factors

## Abstract

**Objective:**

To determine the impacts of climate variability on foodborne diarrhoeal disease worldwide.

**Methods:**

This work was performed based on PRISMA guideline. Articles were retrieved from the PubMed, MEDLINE, Web of Science, Scopus, DOAJ, and Google Scholar. The search was made using Boolean logic operators, medical subject headings, and main keywords related to foodborne diarrheal disease. STATA version 17 was used to perform an analysis. The quality of the articles was evaluated using Joanna Briggs Institute appraisal tools.

**Results:**

The present study included 54 articles with an estimates of 103 findings. An increases in temperature, relative humidity, precipitation, rainfall, and flooding were associated with 4% [RR: 1.04; 95% CI: 1.03, 1.05], 3% [RR: 1.03; 95% CI: 1.01, 1.06], 2% [RR: 1.02; 95% CI: 1.01, 1.03], 1% [RR: 1.01; 95% CI: 1.00, 1.02], and 42% [RR: 1.42; 95% CI: 1.26, 1.57] increases in foodborne diarrhoeal disease, respectively.

**Conclusion:**

There was a significant association between foodborne diarrhoeal disease and climate variability, and indicate the need for building a climate-resilient food safety system to reduce foodborne diarrheal disease.

**Systematic Review Registration:**

identifier CRD42024532430.

## Introduction

Foodborne diseases constitute one of the major causes of mortality and morbidity worldwide, even though they are common in developing countries [1]. Among foodborne disease, foodborne diarrheal disease is common and imposes significant health and economic burdens across the world, particularly in the African and Southeast Asian regions [2]. According to the World Health Organization (WHO) estimates of foodborne disease, there were approximately 600 million cases of foodborne illness globally in 2010, resulting in approximately 33 million disability-adjusted life years (DALYs), of which 550 million were due to diarrheal diseases caused mainly by *norovirus*, *Campylobacter* spp., *Vibrio cholerae*, *Shigella* spp., enteropathogenic *Escherichia coli* (*E. coli),* and enterohemorrhagic *E. coli* [3].

According to the European Food Safety Authority (EFSA) report, the overall incidence of diarrheal per 100,000 people was highest for *Campylobacter* (19.5), followed by *Salmonella* (17.1), *Shigella* (4.8), *Cyclospora* (1.5), *Yersinia* (1.4), *Vibrio* (0.9), and *Listeria* (0.3) [4]. In Africa alone, approximately 91 million people become sick, and 137,000 die annually [1].

These problems occurred not only in lower-income countries but also in higher-income countries, including Europe, which reported 41–49 DALYs per 100,000 people attributable to foodborne disease [5]. Climate variability is considered a serious global challenge influencing the growth and survival of different pathogens that cause food- and water-borne diarrheal diseases and their transmission pathways [6].

Climate variability such as long-term changes in temperature, humidity, rainfall patterns, and extreme weather affect food safety throughout the food chain, including during farming, and they can also affect the nutritional quality of food by influencing the occurrence and intensity of foodborne diseases, particularly foodborne diarrheal diseases [4]. Many foodborne pathogens are zoonotic in nature, and are the major cause of foodborne diarrheal disease; therefore, there is a need for the integration of public health and veterinary communities for early disease detection and control of pathogens in food [7].

There is a need for precise information on the burden of foodborne diseases, particularly foodborne diarrheal diseases which can adequately inform policymakers and help allocate appropriate resources for food safety control and intervention efforts [3]. Because climate change is resulting in increased extreme weather and the emergence or re-emergence of pathogenic microorganisms, the integration of these factors into risk-based approaches for surveillance and response is an important element of improved preparedness [8].

Until this review was conducted, there is no study has provided comprehensive evidence regarding the impacts of various climate variability on foodborne diarrheal disease worldwide. Some of the previous studies reported regional-based evidence on foodborne diseases [9, 10] while another studies were not reported quantitative outcomes, particularly of associations between Climate variability and foodborne diarrheal disease [9–12], whereas other studies have been conducted on single pathogenic bacteria [13]. This indicate there is a need to provide a comprehensive pooled evidence which is necessary for effective intervention, particularly foodborne diarrheal disease associated with climate variability. Therefore, this review aimed to present the impacts of different climate variability on foodborne diarrheal disease.

## Methods

### Protocol and Registration

The current systematic review and meta-analysis was performed under the Preferred Reporting Items for Systematic Review and Meta-Analysis (PRISMA) protocols and guidelines. This review protocol is registered on PROSPERO, with a registration code of CRD42024532430.

### Eligibility Criteria

#### Inclusion Criteria

• **Population**: Studies conducted on all age groups.
• **Outcome**: studies that reported quantitative outcomes (relative risk, risk ratio, and hazard ratio with a 95% confidence interval), particularly those that presented the associations between foodborne diarrhoeal diseases and temperature, relative humidity, rainfall, precipitation, and flooding. This review included studies conducted on any type of foodborne diarrheal disease, regardless of the type of foodborne diarrheal disease.
• **Intervention or exposure reviewed**: Foodborne diarrheal disease and climate variability
• **Types of Articles**: full-text, peer-reviewed, and published articles, particularly those written in English.
• **Publication and survey year**: there was no limitation in terms of publication or survey year.
• **Regions**: studies conducted in any region or country around the world.
• **Exclusion criteria:** Review articles, reports, commentaries, editorial papers, short communications, case studies, preprints, theses and dissertations, and articles with a high risk of bias were excluded from the current review

### Information Sources and Search Strategies

The authors (DAM and TG) retrieved articles from the following databases and websites: PubMed, MEDLINE, Web of Science, Scopus, Cochrane Library, CINAHL, DOAJ, and Google Scholar from 8 April 2024, to 25 April 2024. The authors (DAM and TG) used a combination of Boolean logic operators (AND, OR, and NOT), medical subject headings (MeSH), and main keywords such as climate change, foodborne disease, *salmonellosis, shigellosis*, dysentery diarrhea, *listeriosis*, *Campylobacter* infection, temperature, relative humidity, rainfall, precipitation, flooding, and extreme events, particularly to retrieve articles from the included data sources. Furthermore, the reference lists of the included articles were further screened for additional articles. The search strategies employed in this study are available as Supplementary Material (Supplementary Material S1).

### Study Selection Process

A PRISMA flow chart was used for the selection process of studies included in the current review. The number of articles included in and excluded from the study is presented in the PRISMA flow chart, with the reasons for exclusion. The authors used Endnote (Thomson Reuters, United States) to remove duplicate articles. The authors independently screened and evaluated the articles to determine their eligibility. Disagreements made in the selection process, were resolved by discussion. Finally, those articles that met the inclusion criteria and were eligible for inclusion were included in the current review.

### Quality Assessment

In the present study, the quality of the studies was evaluated using the Joanna Briggs Institute Critical Assessment Tool (JBI) [14]. This tool contains nine evaluation criteria (Supplementary Material S2). Each evaluation criterion parameter was given a value of one if it met the criteria and zero if it did not. On the basis of the total score obtained from these nine evaluation criteria, each article was categorized as low, moderate, or high quality; those articles scored 60% or less, 60%–85%, and 85% and above, respectively. Finally, those articles of moderate or high quality were included in this study. Disagreements between the authors during the quality assessment were solved by discussion and repeating the same procedures.

### Data Extraction

The authors extracted the data using Microsoft Excel (developed by the authors). The following data were extracted from the included articles: authors, sample size, survey year, publication year, region or countries where the study was conducted, target group or study population, types of Climate variability or climatic factors (temperature, relative humidity, rainfall, flooding and precipitation), and their associations with foodborne diarrheal disease, including salmonellosis, *Escherichia coli* infection, dysentery diarrhea (shigellosis), *Campylobacter* infection, *hepatitis* A, *norovirus*, and *rotavirus* infections.

### Statistical Procedures and Data Analysis

The pooled estimate of the associations between foodborne diarrhoeal disease and climate variability, particularly temperature, relative humidity, rainfall, flooding, and precipitation, was performed via STATA version 17 statistical software. The pooled estimate of the associations between foodborne diarrhoeal disease and temperature, relative humidity, rainfall, flooding, and precipitation. Finally, the data were visualized and presented via a random-effects forest plot.

The heterogeneity of the articles was evaluated using the I-square test (I^2^ statistic). The level of heterogeneity is presented as no significant heterogeneity (0%–25%), low heterogeneity (25%–50%), moderate heterogeneity (50%–75%), or high heterogeneity (>75%) [15]. The publication bias was assessed using the funnel plots. Subgroup analysis was performed based on the study population or target group to determine the pooled estimate among different to assess the potential explanation for heterogeneity.

### Sensitivity Analysis

Sensitivity analysis was performed by excluding one or the highest outcome expected to influence the overall estimate of an association between foodborne diarrheal disease, and temperature, relative humidity, rainfall, flooding, and precipitation.

## Results

### Study Selection Process

The authors (DAM and TG) retrieved 2,981 articles from the electronic databases and websites (PubMed, Web of Science, Medline, Science Direct, and Google Scholar as well as screening of references from the eligible articles). A total of 1791 duplicate articles were excluded. Then, 1,190 articles were evaluated on the basis of their title followed by their abstract, of which 642 were excluded because they were unrelated titles and research areas.

Furthermore, 548 articles were further evaluated on the basis of their full text, of which 109 were not available in the full text. Finally, 611 articles were evaluated on the basis of their objectives, methods, and outcomes of interest. Finally, 54 articles, with 103 estimates, that reported an association between different factors and foodborne diarrheal disease were included in the current study (Supplementary Material S3).

### General Characteristics of the Included Articles

In the present study, 36 [2, 16–50] studies, with 49 estimates, reported an association between temperature and foodborne diarrheal disease, of which 6 articles reported more than one outcome. The estimates ranged from RR: 0.98, 95% CI: 0.97, 0.99 in Vietnam [46] to RR: 1.21:95% CI: 1.09, 1.34 in Spain [47]. Among the included articles, 36 focused on all age groups [2, 16, 17, 19–22, 24–30, 32–34, 38, 40–42, 45, 47, 49], whereas 13 focused on children [18, 23, 31, 35–37, 39, 43, 44, 46, 48, 50].

A total of 13 studies [2, 18, 20, 29, 34, 40–43, 51–54], with a total of 15 estimates, reported an association between relative humidity and foodborne diarrheal disease. Among these studies, 10 [2, 20, 29, 34, 40–42, 51, 52, 54] were conducted on all age groups, whereas three studies were conducted on children [18, 43, 53]. Furthermore, 11 studies, with 16 estimates reported an association between precipitation and foodborne diarrheal disease among all age groups [24, 25, 27–29, 40–42, 55–57]. The sample size ranged from 105 study participants in the USA [56] to 7315738 in Mozambique [27].

Furthermore, a total of 12 articles reported an association between rainfall and foodborne diarrheal disease [20, 21, 23, 30, 34, 36, 43–45, 48, 50, 58], with the number of study participants ranging from 461 in India [36] to 1,483,316 in Bhutan [45]. Among these studies, six were conducted on all age groups [20, 21, 30, 34, 45, 58], whereas seven were conducted on children [23, 36, 43, 44, 48, 50]. In the present study, 10 articles reported an association between flooding events and foodborne diarrheal disease [59–68], with the number of study participants ranging from 2,852 in Bangladesh [66] to 359,580 in China [68] (Table 1).

**TABLE 1:** General characteristics of the studies reporting the impacts of different climatic factors or climate variability on foodborne diarrheal disease, worldwide, 2024 (54 articles: 103 estimates).

References	Sample size	Survey year	Target group	Foodborne diarrheal disease	Outcome: RR:95%CI	Location	Quality
[46]	58,773	2005 to 2010	Children	Diarrhea	T^o^:0.98: 0.97, 0.99	Vietnam	High
[51]	3,115	1996 to 2001	All age	Rotavirus	Rh:1.026:1.00·0, 1.053	Bangladesh	High
[52]	569	2008 to 2018	All age	Salmonellosis	Rh: 1.03:1.02–1.05	Iran	High
[53]	423,142	2000 to 2008	Children	Gastroenteritis	Rh:1.039: 2·8, 5·0	Japan	Medium
[42]	2,186	2018 to 2020	All age	Diarrhea	Rh:1.0213: 1.0179, 1.0247	Indonesia	High
[42]	1,246	2018 to 2020	All age	Diarrhea	Rh:1.0166: Rh:1.0151, 1.0181	Indonesia	High
[54]	167,691	2006 to 2017	All age	Diarrhea	Rh:1.23: 1.21–1.25	China	High
[48]	219,774	2003–2013	Children	Diarrhea	T^o^:1.081: 1.02–1.14, Rf: 1.009: 1.004, 1.015	Nepal	High
[47]	275,182	1997 to 2013	All age	Gastroenteritis	T^o^:1.21: 1.09, 1.34	Spain	High
[45]	1,483,316	2003 to 2013	All age	Diarrhea	T^o^: 1.006: 1.005, 0.6, Rf: 1.05: 1.049, 1.051	Bhutan	High
[16]	29,762	1999 to 2010	All age	*Campylobacter* jejuni	T^o^: 1.161: 1.072, 1.249	Israel	High
[16]	29,762	1999 to 2010	All age	*Campylobacter* coli	T^o^: 1.188:1.048, 1.328	Israel	High
[17]	5,040	1991 to 2001	All age	Salmonellosis	T^o^: 1.102:1.087, 1.116	Australia	Medium
[17]	7,212	1991 to 2001	All age	Salmonellosis	T^o^: 1.056:1.043, 1.07	Australia	Medium
[17]	3,973	1991 to 2001	All age	Salmonellosis	T^o^: 1.049: 1.03, 1.064	Australia	Medium
[17]	7,155	1991 to 2001	All age	Salmonellosis	T^o^: 1.051:1.038, 1.065	Australia	Medium
[17]	7,272	1991 to 2001	All age	Salmonellosis	T^o^: 1.041:1.031, 1.052	Australia	Medium
[2]	1,798,198	2005 to 2018	All age	Diarrhea	T^o^: 1.013:0.998, 1.027, Rh: 1.030:1.004, 1.057	Singapore	High
[18]	57,331	1993 to 1998	Children	Diarrhea	T^o^: 1·08: 1·07, 1·09, Rh: 0·97: 0·97, 0·98	Peru	Medium
[19]	12,717	2006 to 2012	All age	Bacillary dysentery	T^o^: 1·04: 1·00, 1·07	China	High
[20]	11,324	2005 to 2015	All age	Salmonellosis	T^o^: 1.043:1.003, 1.084, Rh: 0.987:0.981, 0.994, Rf: 1.008: 1.002, 1.015	Singapore	High
[21]	13,069	2003 to 2006	All age	Cholera cases	T^o^: 1.052: 1.04, 1.06, Rf: 1.025: 1.01–1.04	Zambia	High
[49]	6282	1992 to 2000	All age	Salmonellosis	T^o^: 1.012: 1.009, 1.015	Canada	High
[49]	1743	1992 to 2000	All age	*Campylobacter* infection	T^o^: 1.022: 1.019, 1.024	Canada	High
[49]	9,664	1992 to 2000	All age	*E. Coli* case	T^o^: 1.06: 1.05, 1.069	Canada	High
[49]	986	1992 to 2000	All age	*Campylobacter* cases	T^o^: 1.045: 1.033, 1.058	Canada	High
[22]	2,983,850	1981 to 2010	All age	Diarrhea	T^o^: 1.049: 1.036.1.062	Bangladesh	High
[23]	22,515	2010 to 2017	Children	Diarrhea	T^o^: 1.1666: 1.164–1.168, Rf: 1.00167: 1.00131,1.00193	Ethiopia	High
[24]	4,585	1992 to 2008	All age	Salmonellosis	T^o^: 1.0232:1.0038, 1.0427, Pr: 1.0024: 1.0002, 1.0046	Russia	High
[25]	2,180	2000 to 2010	All age	Salmonellosis	T^o^: 1.055:1.022, 1.088, Pr: 1.005:1.001, 1.01	Kazakhstan	High
[25]	6323	2000 to 2010	All age	Salmonellosis	T^o^: 1.015:1.027, 1.058, Pr: 1.001:1.003, 1.004	Kazakhstan	High
[25]	928	2000 to 2010	All age	Salmonellosis	T^o^: 1.00: 1.031, 1.029, Pr: 1.001:1.006, 1.008	Kazakhstan	High
[25]	1,006	2000 to 2010	All age	Salmonellosis	T^o^: 1.035:1.021, 1.09, Pr: 1.001:1.006, 1.035	Kazakhstan	High
[26]	536	2001 to 2002	All age	Gastroenteritis	T^o^: 1.0248: 1.0101, 1.039	Australia	Medium
[27]	7,315,738	1997 to 2014	All age	Diarrhea	T^o^: 1.0364: 1.0335, 1.0393, Pr: 1.0104: 1.0042, 1.0166	Mozambique	High
[28]	9,529	2002–2012	All age	Salmonellosis	T^o^: 1.041: 1.013, 1.069, Pr: 1.056:1.035, 1.078	USA	High
[29]	596,343	1999 to 2013	All age	Shigellosis	T^o^: 1.06: 1.04, 1.09, Rh: 1.01: 1, 1.01, Pr: 1.04: 1.01, 1.07	Vietnam	High
[30]	142,065	2007 to 2012	All age	Bacillary dysentery	T^o^: 1.0106:1.0063, 1.0149, Rf: 1.0022: 1.0012, 1.0032	China	High
[31]	6511	2006 to 2012	Children	Bacillary dysentery	T^o^: 1.0158: 1.0046, 1.0271	China	High
[32]	395,321	2014 to 2016	All age	Bacillary dysentery	T^o^: 1.017: 1.012, 1.021	China	High
[38]	35,601	1990 to 2012	All age	*Campylobacter* cases	T^o^: 0.995: 0.993, 0.997	Australia	High
[33]	7,845	1990 to 2012	All age	Salmonellosis	T^o^: 1·013: 1·008, 1·019	Australia	High
[34]	136,694	2004 to 2011	All age	Diarrhea	T^o^: 1.07: 1.04–1.08, Rh: 1.13: 1.12, 1.15, Rf: 1.05:1.05–1.08	Vietnam	High
[35]	25,385	2013 to 2017	Children	Diarrhea	T^o^: 1.046: 1.007, 1.088	Bangladesh	High
[36]	461	2017–2019	Children	Diarrhea	T^o^: 1.0397: 1.0292, 1.0502, Rf: 1.0012: 1.0017, 1.0008	India	High
[37]	29,639	2010 to 2012	Children	Bacillary dysentery	T^o^: 1.113:1.1047, 1.1212	China	High
[39]	11,194	2003 to 2009	Children	Diarrhea	T^o^: 1.03: 1.02, 1.05	Australia	High
[40]	44,926	2010 to 2015	All age	Bacillary dysentery	T^o^: 1.032: 1.024, 1.041, Rh: 1.007:1.001, 1.013, Pr: 1.01; 0.9997, 1.0003	China	High
[41]	710,202	2013 to 2017	All age	Bacillary dysentery	T^o^: 1.01: 1.00, 1.02, Rh: 0.998:0.99, 1.00, Pr: 1.0101: 1.003, 1.019	China	High
[41]	710,202	2013 to 2017	All age	Bacillary dysentery	T^o^: 1.04:1.03, 1.05, Rh: 1.00: 0.99, 1.01, Pr: 1.001:1.00, 1.01	China	High
[42]	4,117	2018 to 2020	All age	Diarrhea	T^o^: 1.0539: 1.0461, 1.0617, Pr: 1.0113: 1.0102, 1.0124	Indonesia	High
[43]	97,918	2017–2020	Children	Diarrhea	T^o^: 1.103: 1.009, 1.206, Rh: 0.973: 0.953, 0.994, Pr: 1.0305: 2.09, 4.01, Rf: 0.999:0.999, 1.000	Indonesia	Medium
[50]	8,309	2002 to 2011	Children	Rotavirus	T^o^: 1.0332: T^o^: 1.026.1.0424, Rf: 1.004: 1.002, 1.0079	China	High
[50]	3,928	2002 to 2011	Children	Norovirus	T^o^: 1.0234: T^o^: 1.0152, 1.0358, Rf: 1.0193:1.0121, 1.0309	China	High
[44]	217,734	2013 to 2015	Children	Diarrhea	T^o^: 1.019:1.0034, 1.0347, Rf: 1.0004:1.0001, 1.0007	Ethiopia	High
[55]	6243	2016 to 2020	All age	Hepatitis A	Pr: 0.97: 0.94, 1.00	Korea	Medium
[56]	105	2015 to 2016	All age	Shigellosis	Pr: 1.18: 1.06, 1.33	USA	High
[57]	14,800	2004 to 2013	All age	Salmonellosis	Pr: 1.146: 1.092, 1.203	Australia	High
[58]	33,927	2013 to 2014	All age	Diarrhea	Rf: 1.35: 1.14–1.60	Ecuador	Medium
[64]	18,976	2004 to 2010	All age	Bacillary Dysentery	Fl: 1.17: 1.03–1.33	China	High
[62]	24,536	2004 to 2009	All age	Dysentery	Fl: 1.66: 1.52, 1.82	China	High
[61]	45,131	2006 to 2010	All age	Diarrhea	Fl: 1.24: 1.11–1.40	China	High
[60]	9,255	2004 to 2010	All age	Bacillary dysentery	Fl: 1.78 : 1.61, 1.97	China	High
[59]	4,812	2005 to 2011	All age	Bacillary dysentery	Fl: 1.29: 1.14, 1.46	China	High
[63]	274,621	2013 to 2017	All age	Diarrhea	Fl: 1.11: 1.01, 1.23	China	High
[67]	45,691	2005 to 2016	All age	Bacillary dysentery	Fl: 1.393:1.216, 1.596	China	High
[65]	7,591	2004 to 2010	All age	Dysentery	Fl: 1.74:1.56, 1.94	China	High
[66]	2,852	2001 to 2007	All age	Diarrhea	Fl: 1.55: 1.12, 2.15	Bangladesh	High
[68]	359,580	2013 to 2017	All age	Diarrhea	Fl: 1.29: 1.15, 1.46	China	High

Keys: Fl, Flooding; Rf, Rain Fail; Pr, precipitation; Rf, Relative humidity; RR, relative risk; CI, confidence interval; T^o^, Temperature.

#### Associations Between Temperature and Foodborne Diarrhoeal Disease

**FIGURE 1:**
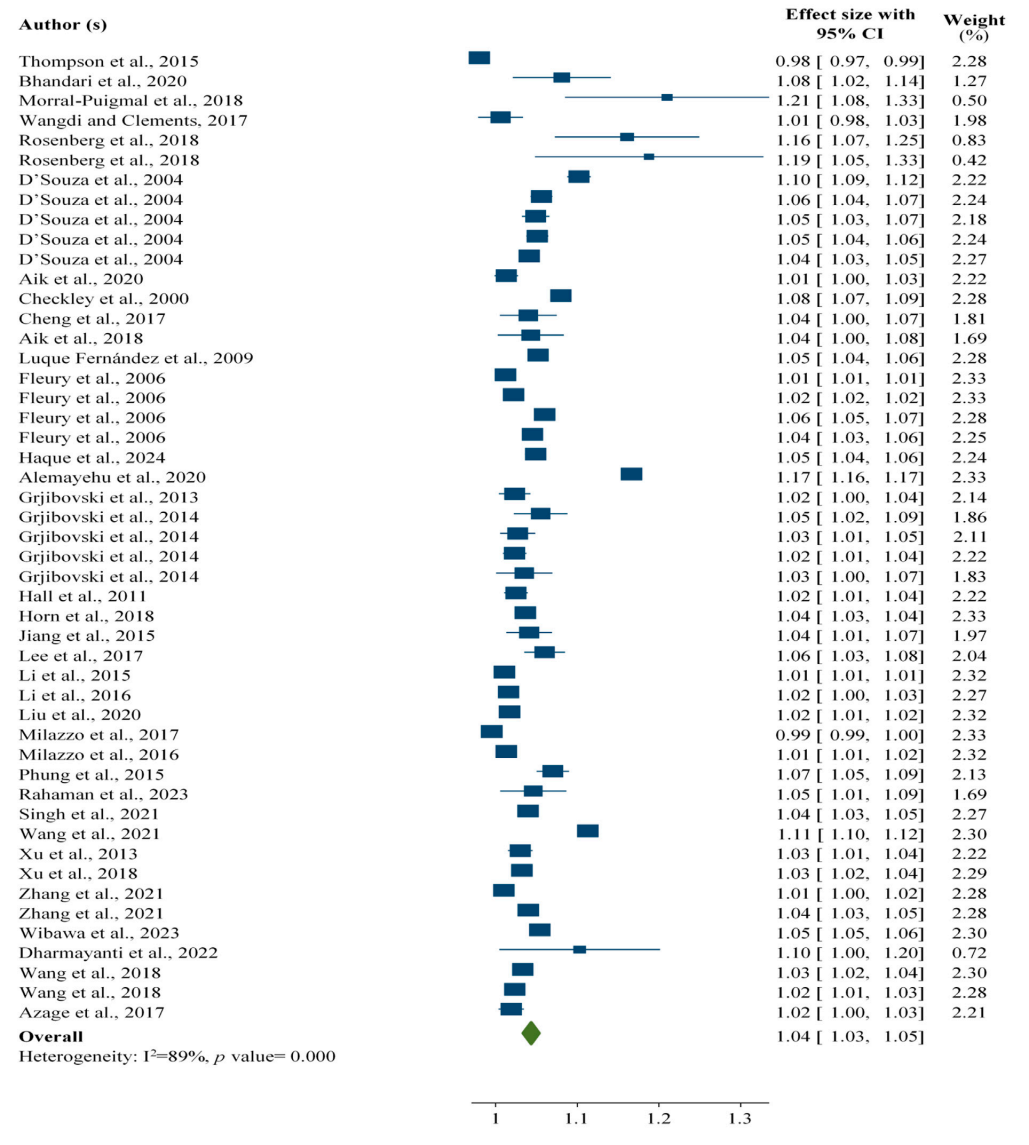
Association between temperature and foodborne diarrheal disease among the study participants, worldwide, 2024.

The current study revealed that every 1°C increase in temperature is associated with a 4% (RR: 1.04; 95% CI: 1.03, 1.05) increase in the number of foodborne diarrheal disease worldwide, regardless of the age and type of foodborne diarrheal disease reported in the studies included in the study (Figure 1).

On the basis of the subgroup analyses by the age group of the study participants, the evidence from the current findings revealed that every 1°C increase in temperature was associated with a 4% [RR: 1.04; 95% CI: 1.03, 1.04) increase in the number of foodborne diarrheal disease cases among all age groups, whereas it accounted for a 6% [RR: 1.06; 95% CI: 1.01, 1.1] increase in foodborne diarrheal disease among children across the world. The total increase in the number of foodborne diarrhoeal cases after the subgroup analysis was 5% [RR: 1.05; 95% CI: 1.03, 1.07] for every 1°C increase in temperature (Supplementary Material S4).

Furthermore, to determine the effects of extreme values expected to affect the pooled outcome, four extreme findings were removed. After four findings were removed, a 1°C rise in temperature was associated with a 4% [RR: 1.04; 95% CI: 1.03, 1.05) rise in foodborne diarrhoeal disease (Supplementary Material S5).

#### Associations Between Relative Humidity and Foodborne Diarrhoeal Disease

**FIGURE 2:**
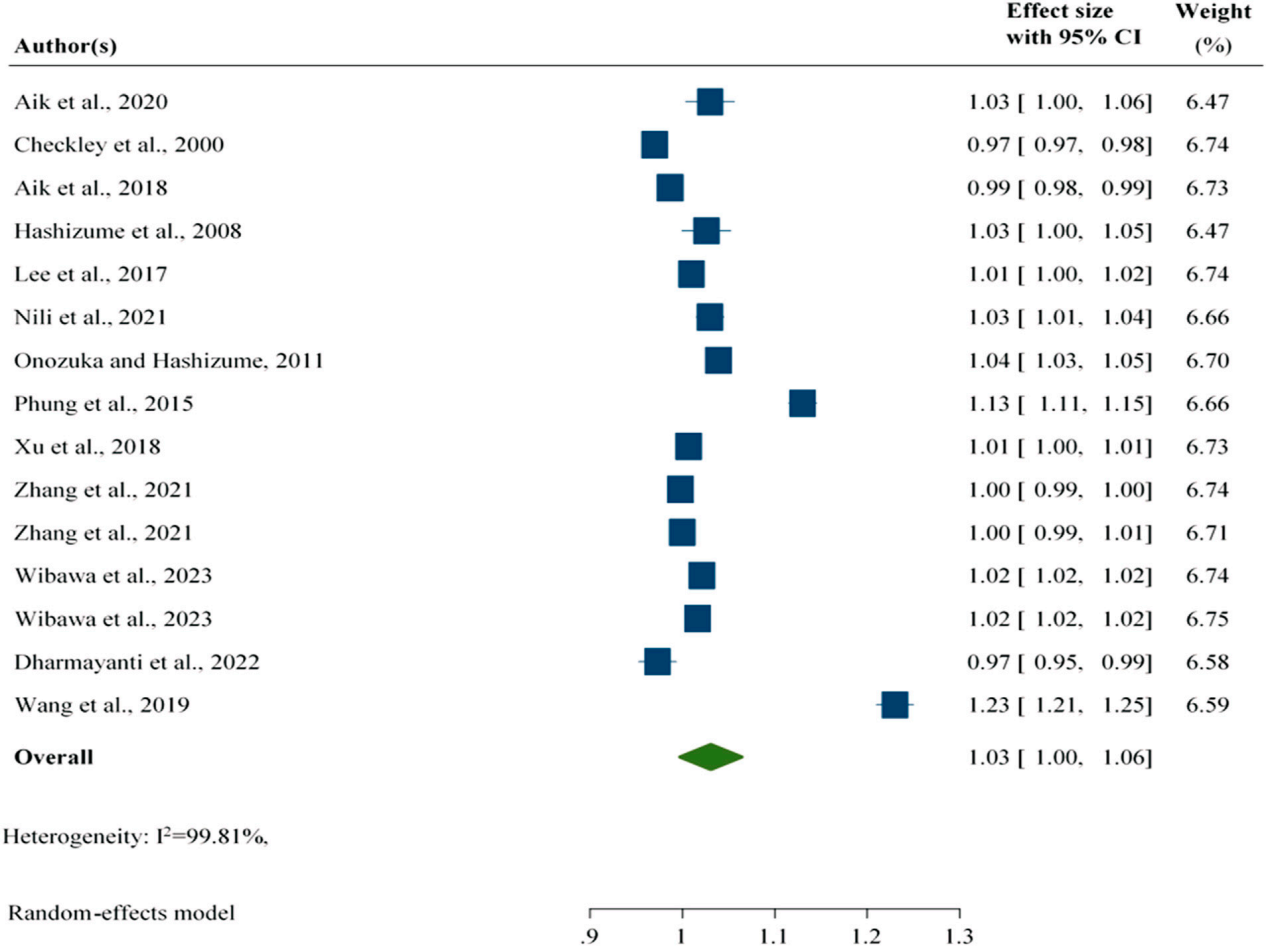
Association between relative humidity and foodborne diarrheal disease regardless of the study group, worldwide, 2024.

This study revealed that an increase in relative humidity was associated with a 3% [RR: 1.03; 95% CI: 1.01, 1.06] increase in the number of foodborne diarrheal disease cases worldwide, regardless of the age and type of foodborne diarrheal disease reported in the studies included in the study (Figure 2).

The subgroup analysis findings revealed that an increase in relative humidity was associated with a 4% [RR: 1.04; 95% CI: 1.01, 1.08] increase in the number of foodborne diarrhoeal disease cases among all ages. Furthermore, an increase in relative humidity was associated with a lower number of foodborne diarrhoeal cases among children [RR: 0.99; 95% CI: 0.95, 1.04]. However, the overall evidence after subgroup analysis revealed that an increase in relative humidity was associated with a 3% [RR: 1.03; 95% CI: 1.01, 1.06] increase in the number of foodborne diarrhoeal disease cases, which is similar to the findings of a previous subgroup analysis (Supplementary Material S6).

After the two largest outcomes were excluded from the analysis, an increase in relative humidity was associated with a 2% [RR: 1.02; 95% CI: 1.00, 1.02] increase in the number of foodborne diarrheal disease cases (Supplementary Material S7).

#### Associations Between Precipitation and Foodborne Diarrhoeal Disease

**FIGURE 3:**
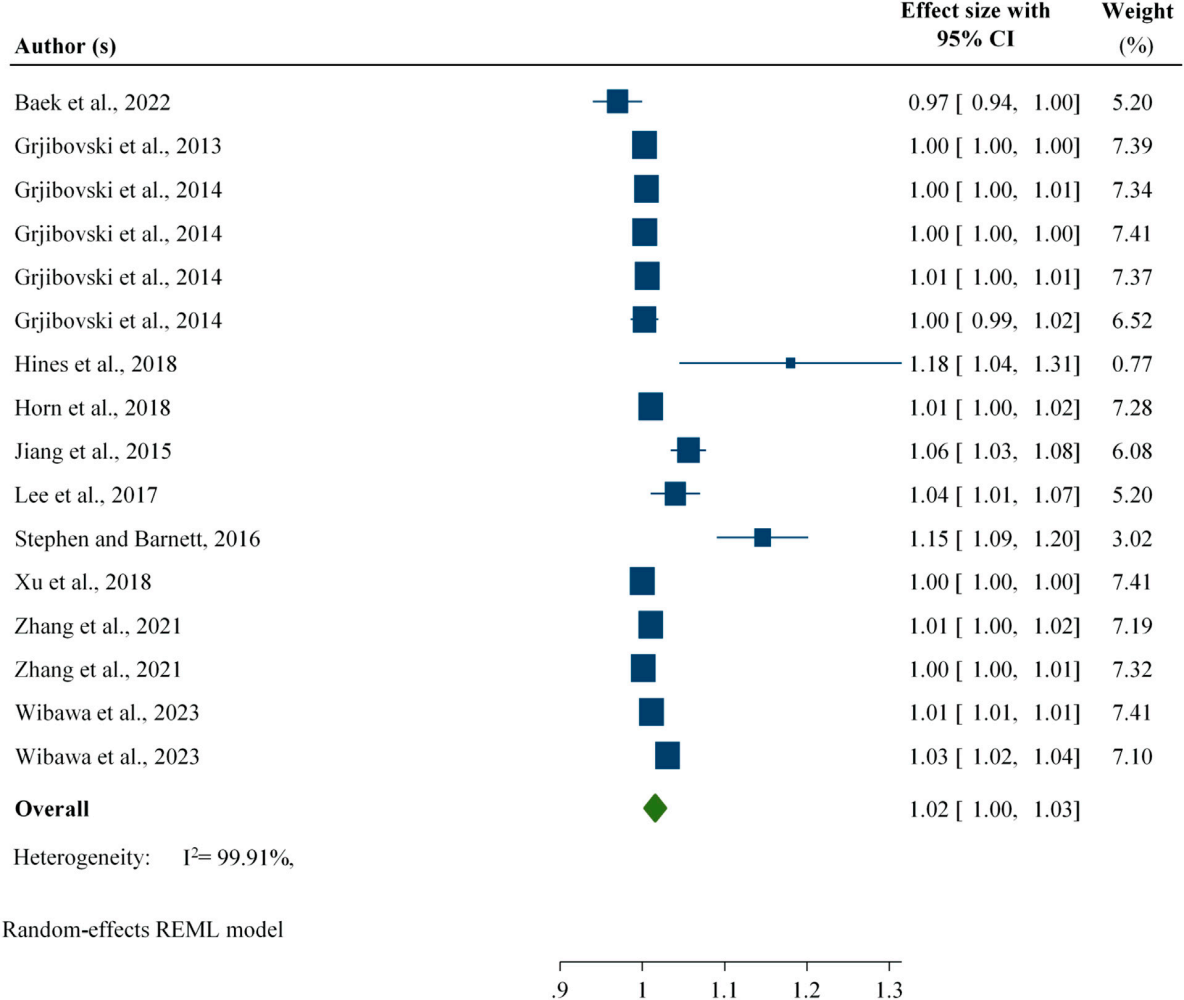
Association between foodborne diarrhoeal disease and precipitation regardless of age group, worldwide, 2024.

An increase in precipitation is associated with a 2% [RR: 1.02; 95% CI: 1.01, 1.03] increase in the number of foodborne diarrheal disease cases across the world, regardless of the age groups and types of diarrheal disease reported by the studies included in the study (Figure 3).

Furthermore, to determine the effects of extreme values expected to affect the pooled outcome, four findings were removed. After four findings were removed, an increase in precipitation was associated with a 1% [RR: 1.01; 95% CI: 1.00, 1.02] increase in diarrheal disease (Supplementary Material S8).

#### Associations Between Rainfall and Foodborne Diarrhoeal Disease

**FIGURE 4:**
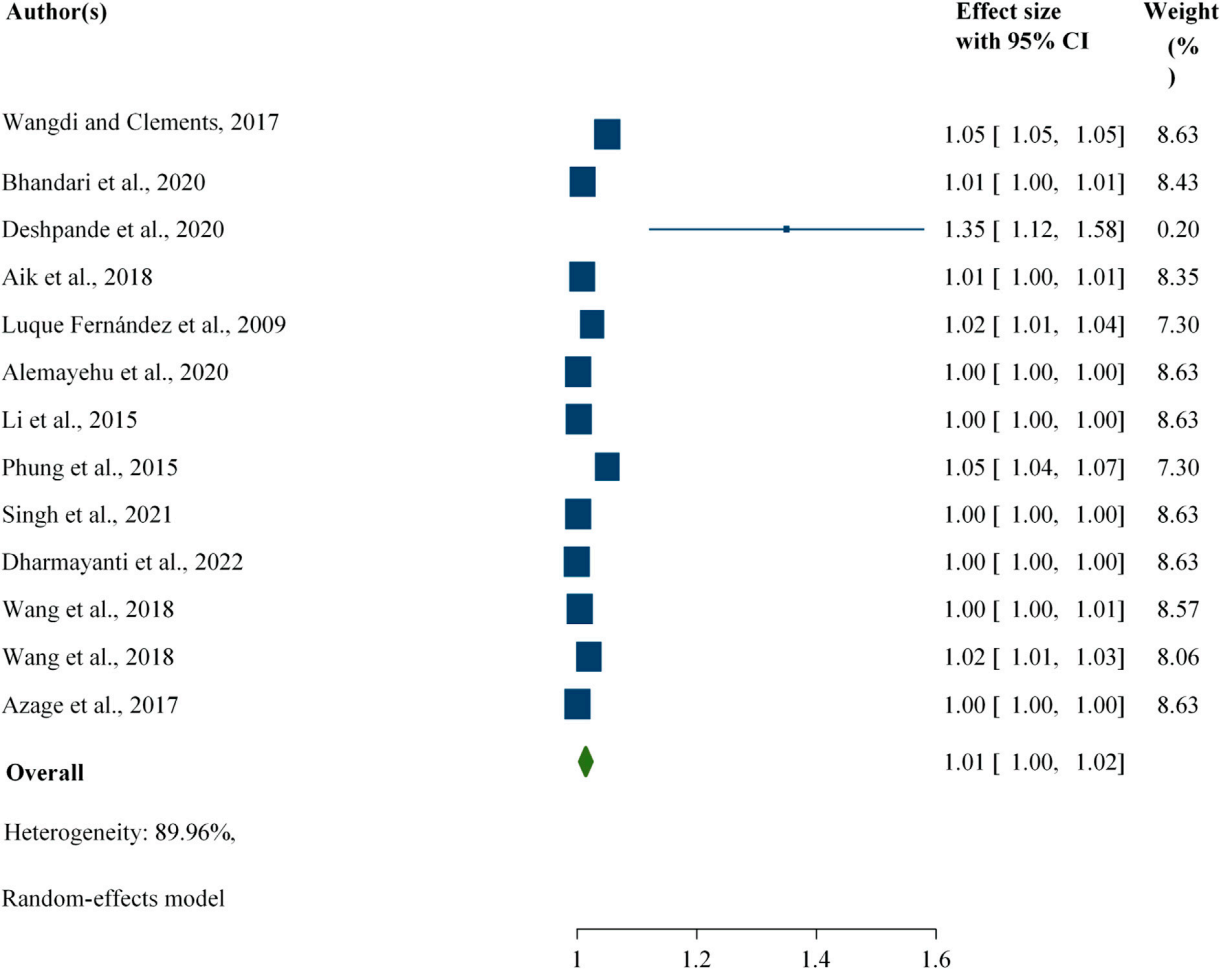
Association between rainfall and foodborne diarrheal disease among the study participants, worldwide, 2024.

The evidence from 13 estimates revealed that an increase in rainfall was associated with a 1% [RR: 1.01; 95% CI: 1.00, 1.02] increase in foodborne diarrheal disease, regardless of the study participants (Figure 4).

This study revealed that an increase in rainfall was associated with a 3% [RR: 1.03; 95% CI: 1.01, 1.05) increase in the number of foodborne diarrheal disease cases among all age groups, whereas it accounted for 1% [RR: 1.01; 95% CI: 1.00, 1.01) among the children (Supplementary Material S9).

After one largest outcome was excluded from the analysis, the study revealed a similar association before excluding the largest outcome, which was expected to affect the pooled evidence [RR: 1.01; 95% CI: 1.00, 1.02) (Supplementary Material S10).

#### Associations Between Flooding and Foodborne Diarrhoeal Disease

**FIGURE 5:**
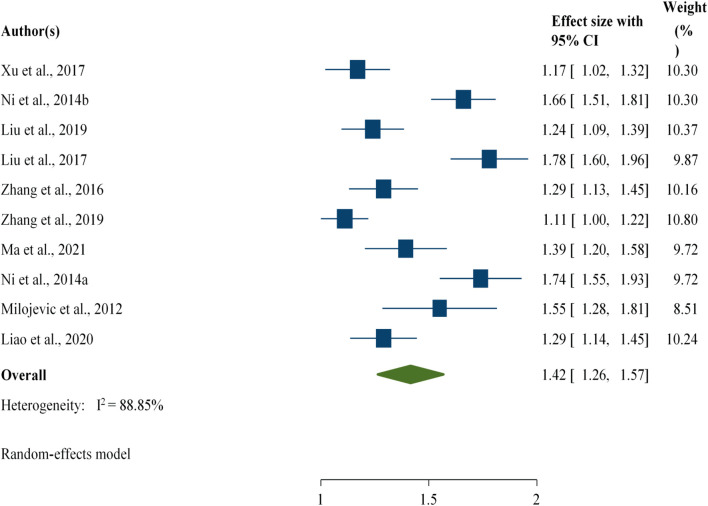
Association between flooding and foodborne diarrheal disease among the study participants, worldwide, 2024.

The current study revealed that an increase in flooding events was associated with a 42% [RR: 1.42; 95% CI: 1.26, 1.57] increase in diarrhoeal disease cases, regardless of the study group (Figure 5).

## Discussion

The current study revealed that an increase in temperature is associated with a 4% [RR: 1.04; 95% CI: 1.03, 1.05) increase in the number of diarrhoeal disease cases across the world, regardless of the age and group of the study participants. The finding of the current study is supported by another meta-analysis, which reported a 7% (RR: 1.07; 95% CI: 1.03, 1.10) increase in diarrheal diseases among all cases in developing countries [69], this study revealed that the incidence of foodborne diarrheal disease was greater among children [6% (RR: 1.06; 95% CI: 1.01, 1.1)] than among all age groups [4% (RR: 1.04; 95% CI: 1.03, 1.043)].

An increase in relative humidity was associated with a 3% [RR: 1.03; 95% CI: 1.01, 1.06] increase in the number of foodborne diarrheal disease cases worldwide. However, an increase in relative humidity was associated with a lower number of foodborne diarrhoeal cases among children [RR: 0.99; 95% CI: 0.95, 1.04]. Furthermore, after two estimates with the highest outcome were excluded from the analysis, particularly to assess the influence of extreme outcomes on the pooled estimate, an increase in relative humidity was associated with a 2% [RR: 1.02; 95% CI: 1.00, 1.02] increase in the number of foodborne diarrheal disease cases, which indicates no potential impacts of extreme outcomes on the pooled estimate.

An increase in precipitation was associated with a 2% [RR: 1.02; 95% CI: 1.01, 1.03] increase in the number of foodborne diarrheal disease cases worldwide, regardless of the target population. In addition, to determine the effect of an extreme outcome on the pooled estimate, the data were analysed by excluding the estimate with the highest value, and an increase in precipitation was associated with a 1% [RR: 1.01; 95% CI: 1.00, 1.02] increase in foodborne diarrheal disease, which was relatively lower than the pooled finding before an extreme outcome was excluded. However, there was a significant association.

According to this study, an increase in rainfall was associated with a 1% [RR: 1.01; 95% CI: 1.00, 1.02] increase in foodborne diarrhoeal disease, regardless of the target population. This finding is supported by another study that reported an association between a rise in extreme rain events and increased incidence of diarrhoeal disease (IRR: 1.26; 95% CI: 1.05, 1.51) [70]. The present study revealed a lower incidence of foodborne diarrhoeal disease, which may be attributed to the variation in the scope of the study, outcome, and geographical location. Because the current study was conducted across the world, it focused particularly on foodborne diarrheal disease. Unlike the association between temperature and foodborne diarrhoeal disease, a higher incidence of foodborne diarrhoeal disease was reported among all age groups [RR: 1.03; 95% CI: 1.01, 1.05) than among children (RR: 1.01; 95% CI: 1.00, 1.01).

In addition, the current study revealed that an increase in flooding events was associated with a 42% [RR: 1.42; 95% CI: 1.26, 1.57] increase in foodborne diarrheal disease cases, regardless of the study group. This finding was supported by a review conducted in China [RR: 1.48; 95% CI: 1.14–1.91] but was slightly greater than the current findings [71]. Furthermore, another review reported a significant association between flooding and the incidence of diarrhea [RR: 1.40, 95% CI: 1.29–1.52] [72]. The variation may be attributed to the difference in their scope in terms of the study region and the number of articles included. Relative humidity, rainfall, and precipitation, flooding events presented major potential impacts on foodborne diarrhoeal disease.

In general, the present study revealed a significant association between foodborne diarrhoeal disease and the following climatic factors or climate variability: temperature, relative humidity, rainfall, precipitation, and flooding. This indicates the need for effective interventions or strategies, particularly for establishing a climate change-resilient food safety system to reduce the health and economic burdens associated with different types of foodborne diarrheal diseases.

### Strengths

This study used multiple databases and websites to retrieve articles regardless of the region where the study was conducted and the publication period. The extracted data were re-entered to avoid errors. The quality of the included articles was assessed via standard quality appraisal tools. Furthermore, this study was conducted according to the PRISMA guidelines for systematic review and meta-analysis.

### Limitations of the Study

There were some limitations, including the unequal distribution of the studies across the world due to the lack of eligible studies and the lack of studies on the impacts of climate variability on foodborne diarrhoeal disease. In addition due to the lack of systematic reviews and meta-analyses conducted in these research areas, the authors compared some review articles with the current findings.

## Conclusions

According to the current study, there were significant associations between foodborne diarrhoeal disease and various climate variability, such as temperature, relative humidity, rainfall, precipitation, and flooding. The prevalence of foodborne diarrhoeal disease associated with climatic factors was greater, particularly for flooding, followed by temperature and relative humidity. In general, the current findings highlight the need for community-based tailored intervention strategies for establishing a climate change-resilient food safety risk management system to reduce the burden of foodborne diarrheal diseases.

## Data Availability

Almost all the data are included in this study, including those in the Supplementary Material. However, some data may be available from the corresponding author upon reasonable request.

## Abbreviations

DOAJ, Directory of Open Access Journals; MeSH, Medical Subject Heading; JBI, Joanna Briggs Institute; PRISMA, Preferred Reporting Items for Systematic Review and Meta-Analysis; WASH, Water, Sanitation, and Hygiene; WHO, World Health Organization.

## References

[1] Cissé G . Foodborne and Water-Borne Diseases under Climate Change in Low-And Middle-Income Countries: Further Efforts Needed for Reducing Environmental Health Exposure Risks. Acta tropica (2019) 194:181–8. 10.1016/j.actatropica.2019.03.012 30946811 PMC7172250

[2] Aik J Ong J Ng L-C . The Effects of Climate Variability and Seasonal Influence on Diarrhoeal Disease in the Tropical City-State of Singapore–A Time-Series Analysis. Int J Hyg Environ Health (2020) 227:113517. 10.1016/j.ijheh.2020.113517 32272437

[3] WHO. World Health Organization. Estimates of the Global Burden of Foodborne Diseases: Foodborne Disease Burden Epidemiology Reference Group 2007-2015. Geneva, Switzerland: World Health Organization (2015). p. 2015.

[4] EFSA. European Food Safety Authority (EFSA); Climate Change and Food Safety (2024). Available from: https://www.efsa.europa.eu/en/topics/topic/climate-change-and-food-safety. (Accessed: March 10, 2024).

[5] Havelaar KT Kirk MD Torgerson PR Gibb HJ Hald T Lake RJ World Health Organization Global Estimates and Regional Comparisons of the Burden of Foodborne Disease in 2010. PLoS Med (2010) 12(12):e1001923. 10.1371/journal.pmed.1001923 PMC4668832 26633896

[6] Anas M Sami MA Siddiqui Z Khatoon K Zeyad MT Malik A . Impact of Climate Change on the Incidence and Transfer of Food-And Water-Borne Diseases. Microbiomes Glob Clim Change (2021) 123–44. 10.1007/978-981-33-4508-9_9

[7] Wielinga PR Schlundt J . One Health and Food Safety. Copenhagen, Denmark: Confronting Emerging Zoonoses: The One Health Paradigm (2014). p. 213–32.

[8] Lake IR . Foodborne Disease and Climate Change in the United Kingdom. Environ Health (2017) 16:117–9. 10.1186/s12940-017-0327-0 29219100 PMC5773878

[9] Amegah AK Rezza G Jaakkola JJ . Temperature-related Morbidity and Mortality in Sub-saharan Africa: A Systematic Review of the Empirical Evidence. Environ Int (2016) 91:133–49. 10.1016/j.envint.2016.02.027 26949867

[10] Arinda D Hidayati R Taufik M . Climate Influence on Diarrhea Disease in Tropical Regions Based on Systematic Literature Review. Agromet (2023) 37(2):99–107. 10.29244/j.agromet.37.2.99-107

[11] Malik I Anjayati S Musdhalifa P Binti D Tosepu R , editors. Impact of Weather and Climate on Diarrhea Incidence: A Review. IOP Conference Series: Earth and Environmental Science. IOP Publishing (2021). 10.1088/1755-1315/755/1/012088

[12] Gao J Sun Y Lu Y Li L . Impact of Ambient Humidity on Child Health: A Systematic Review. PloS one (2014) 9(12):e112508. 10.1371/journal.pone.0112508 25503413 PMC4264743

[13] Philipsborn R Ahmed SM Brosi BJ Levy K . Climatic Drivers of Diarrheagenic *Escherichia coli* Incidence: A Systematic Review and Meta-Analysis. The J Infect Dis (2016) 214(1):6–15. 10.1093/infdis/jiw081 26931446 PMC4907410

[14] JBI. The Joanna Briggs Institute. Critical Appraisal Tools for Use in the JBI Systematic Reviews Checklist for Prevalence Studies. The University of Adelaide (2019). Available from: https://joannabriggs.org/sites/default/files/2019-05/JBI_Critical_AppraisalChecklist_for_Prevalence_Studies2017_0.pdf (Accessed March 12, 2023).

[15] Ades AE Lu G Higgins JP . The Interpretation of Random-Effects Meta-Analysis in Decision Models. Med Decis Making (2005) 25(6):646–54. 10.1177/0272989X05282643 16282215

[16] Rosenberg A Weinberger M Paz S Valinsky L Agmon V Peretz C . Ambient Temperature and Age-Related Notified Campylobacter Infection in Israel: A 12-year Time Series Study. Environ Res (2018) 164:539–45. 10.1016/j.envres.2018.03.017 29609183

[17] D’Souza RM Becker NG Hall G Moodie KB . Does Ambient Temperature Affect Foodborne Disease? Epidemiology (2004) 15(1):86–92. 10.1097/01.ede.0000101021.03453.3e 14712151

[18] Checkley W Epstein LD Gilman RH Figueroa D Cama RI Patz JA Effects of EI Niño and Ambient Temperature on Hospital Admissions for Diarrhoeal Diseases in Peruvian Children. The Lancet (2000) 355(9202):442–50. 10.1016/s0140-6736(00)82010-3 10841124

[19] Cheng J Xie M Zhao K-F Wu J Xu Z Song J Impacts of Ambient Temperature on the Burden of Bacillary Dysentery in Urban and Rural Hefei, China. Epidemiol and Infect (2017) 145(8):1567–76. 10.1017/S0950268817000280 28294081 PMC9203284

[20] Aik J Heywood AE Newall AT Ng L-C Kirk MD Turner R . Climate Variability and Salmonellosis in Singapore–A Time Series Analysis. Sci total Environ (2018) 639:1261–7. 10.1016/j.scitotenv.2018.05.254 29929293

[21] Luque FMÁ Bauernfeind A Jiménez JD Gil CL Omeiri NE Guibert DH . Influence of Temperature and Rainfall on the Evolution of Cholera Epidemics in Lusaka, Zambia, 2003–2006: Analysis of a Time Series. Trans R Soc Trop Med Hyg (2009) 103(2):137–43. 10.1016/j.trstmh.2008.07.017 18783808

[22] Haque F Lampe F Hajat S Stavrianaki K Hasan ST Faruque A Effects of Diurnal Temperature Range on Diarrhea in the Subtropical Megacity of Dhaka, Bangladesh. The J Clim Change Health (2024) 17:100305. 10.1016/j.joclim.2024.100305 PMC12851239 41647661

[23] Alemayehu B Ayele BT Melak F Ambelu A . Exploring the Association between Childhood Diarrhea and Meteorological Factors in Southwestern Ethiopia. Sci The Total Environ (2020) 741:140189. 10.1016/j.scitotenv.2020.140189 32886968

[24] Grjibovski A Bushueva V Boltenkov V Buzinov R Degteva G Yurasova E Climate Variations and Salmonellosis in Northwest Russia: A Time-Series Analysis. Epidemiol and Infect (2013) 141(2):269–76. 10.1017/S0950268812000544 22475326 PMC9152056

[25] Grjibovski A Kosbayeva A Menne B . The Effect of Ambient Air Temperature and Precipitation on Monthly Counts of Salmonellosis in Four Regions of Kazakhstan, Central Asia, in 2000–2010. Epidemiol and Infect (2014) 142(3):608–15. 10.1017/S095026881300157X 23816177 PMC9151133

[26] Hall G Hanigan I Dear K Vally H . The Influence of Weather on Community Gastroenteritis in Australia. Epidemiol and Infect (2011) 139(6):927–36. 10.1017/S0950268810001901 20696089

[27] Horn LM Hajat A Sheppard L Quinn C Colborn J Zermoglio MF Association between Precipitation and Diarrheal Disease in Mozambique. Int J Environ Res Public Health (2018) 15(4):709. 10.3390/ijerph15040709 29642611 PMC5923751

[28] Jiang C Shaw KS Upperman CR Blythe D Mitchell C Murtugudde R Climate Change, Extreme Events and Increased Risk of Salmonellosis in Maryland, USA: Evidence for Coastal Vulnerability. Environ Int (2015) 83:58–62. 10.1016/j.envint.2015.06.006 26093493 PMC6590700

[29] Lee HS Hoang TH Pham-Duc P Lee M Grace D Phung DC Seasonal and Geographical Distribution of Bacillary Dysentery (Shigellosis) and Associated Climate Risk Factors in Kon Tam Province in Vietnam from 1999 to 2013. Infect Dis poverty (2017) 6(03):58–68. 10.1186/s40249-017-0325-z 28637484 PMC5480122

[30] Li Z Zhang X Hou X Xu S Zhang J Song H Nonlinear and Threshold of the Association between Meteorological Factors and Bacillary Dysentery in Beijing, China. Epidemiol and Infect (2015) 143(16):3510–9. 10.1017/S0950268815001156 26027678 PMC9150982

[31] Li K Zhao K Shi L Wen L Yang H Cheng J Daily Temperature Change in Relation to the Risk of Childhood Bacillary Dysentery Among Different Age Groups and Sexes in a Temperate City in China. Public Health (2016) 131:20–6. 10.1016/j.puhe.2015.10.011 26655018

[32] Liu Z Tong MX Xiang J Dear K Wang C Ma W Daily Temperature and Bacillary Dysentery: Estimated Effects, Attributable Risks, and Future Disease Burden in 316 Chinese Cities. Environ Health Perspect (2020) 128(5):057008. 10.1289/EHP5779 32452706 PMC7266621

[33] Milazzo A Giles L Zhang Y Koehler A Hiller J Bi P . The Effect of Temperature on Different Salmonella Serotypes during Warm Seasons in a Mediterranean Climate City, Adelaide, Australia. Epidemiol and Infect (2016) 144(6):1231–40. 10.1017/S0950268815002587 26522685

[34] Phung D Huang C Rutherford S Chu C Wang X Nguyen M Association between Climate Factors and Diarrhoea in a Mekong Delta Area. Int J biometeorology (2015) 59:1321–31. 10.1007/s00484-014-0942-1 25472927

[35] Rahaman MR Dear K Satter SM Tong M Milazzo A Marshall H Short-Term Effects of Climate Variability on Childhood Diarrhoea in Bangladesh: Multi-Site Time-Series Regression Analysis. Int J Environ Res Public Health (2023) 20(13):6279. 10.3390/ijerph20136279 37444126 PMC10341980

[36] Singh N Mall R Banerjee T Gupta A . Association between Climate and Infectious Diseases Among Children in Varanasi City, India: A Prospective Cohort Study. Sci The Total Environ (2021) 796:148769. 10.1016/j.scitotenv.2021.148769 34274660

[37] Wang L Xu C Xiao G Qiao J Zhang C . Spatial Heterogeneity of Bacillary Dysentery and the Impact of Temperature in the Beijing–Tianjin–Hebei Region of China. Int J Biometeorology (2021) 65(11):1919–27. 10.1007/s00484-021-02148-3 34050434

[38] Milazzo A Giles L Zhang Y Koehler A Hiller J Bi P . The Effects of Ambient Temperature and Heatwaves on Daily Campylobacter Cases in Adelaide, Australia, 1990–2012. Epidemiol and Infect (2017) 145(12):2603–10. 10.1017/S095026881700139X 28693637 PMC9148798

[39] Xu Z Huang C Turner LR Su H Qiao Z Tong S . Is Diurnal Temperature Range a Risk Factor for Childhood Diarrhea? PLoS One (2013) 8(5):e64713. 10.1371/journal.pone.0064713 23724083 PMC3665771

[40] Xu C Xiao G Wang J Zhang X Liang J . Spatiotemporal Risk of Bacillary Dysentery and Sensitivity to Meteorological Factors in Hunan Province, China. Int J Environ Res Public Health (2018) 15(1):47. 10.3390/ijerph15010047 PMC5800146 29286297

[41] Zhang X Gu X Wang L Zhou Y Huang Z Xu C Spatiotemporal Variations in the Incidence of Bacillary Dysentery and Long-Term Effects Associated with Meteorological and Socioeconomic Factors in China from 2013 to 2017. Sci Total Environ (2021) 755:142626. 10.1016/j.scitotenv.2020.142626 33039932

[42] Wibawa BSS Maharani AT Andhikaputra G Putri MSA Iswara AP Sapkota A Effects of Ambient Temperature, Relative Humidity, and Precipitation on Diarrhea Incidence in Surabaya. Int J Environ Res Public Health (2023) 20(3):2313. 10.3390/ijerph20032313 36767679 PMC9916310

[43] Dharmayanti I Tjandrarini DH Hidayangsih PS . Climatic Factors and Childhood Diarrhea in South Kalimantan in 2017-2020. Southeast Asian J Trop Med Public Health (2022) 53:65–80.

[44] Azage M Kumie A Worku A Bagtzoglou A C Anagnostou E . Effect of Climatic Variability on Childhood Diarrhea and its High Risk Periods in Northwestern Parts of Ethiopia. PloS one (2017) 12(10):e0186933. 10.1371/journal.pone.0186933 29073259 PMC5658103

[45] Wangdi K Clements AC . Spatial and Temporal Patterns of Diarrhoea in Bhutan 2003–2013. BMC Infect Dis (2017) 17:507–9. 10.1186/s12879-017-2611-6 28732533 PMC5521140

[46] Thompson CN Zelner JL Nhu TDH Phan MV Le PH Thanh HN The Impact of Environmental and Climatic Variation on the Spatiotemporal Trends of Hospitalized Pediatric Diarrhea in Ho Chi Minh City, Vietnam. Health and place (2015) 35:147–54. 10.1016/j.healthplace.2015.08.001 26402922 PMC4664115

[47] Morral-Puigmal C Martínez-Solanas È Villanueva CM Basagaña X . Weather and Gastrointestinal Disease in Spain: A Retrospective Time Series Regression Study. Environ Int (2018) 121:649–57. 10.1016/j.envint.2018.10.003 30316180

[48] Bhandari D Bi P Sherchand JB Dhimal M Hanson-Easey S . Assessing the Effect of Climate Factors on Childhood Diarrhoea Burden in Kathmandu, Nepal. Int J Hyg Environ Health (2020) 223(1):199–206. 10.1016/j.ijheh.2019.09.002 31537454

[49] Fleury M Charron DF Holt JD Allen OB Maarouf AR . A Time Series Analysis of the Relationship of Ambient Temperature and Common Bacterial Enteric Infections in Two Canadian Provinces. Int J biometeorology (2006) 50:385–91. 10.1007/s00484-006-0028-9 16575582

[50] Wang P Goggins WB Chan EY . A Time-Series Study of the Association of Rainfall, Relative Humidity and Ambient Temperature with Hospitalizations for Rotavirus and Norovirus Infection Among Children in Hong Kong. Sci Total Environ (2018) 643:414–22. 10.1016/j.scitotenv.2018.06.189 29940452

[51] Hashizume M Armstrong B Wagatsuma Y Faruque A Hayashi T Sack DA . Rotavirus Infections and Climate Variability in Dhaka, Bangladesh: A Time-Series Analysis. Epidemiol and Infect (2008) 136(9):1281–9. 10.1017/S0950268807009776 17988426 PMC2870922

[52] Nili S Khanjani N Bakhtiari B Jahani Y Dalaei H . The Effect of Meteorological Variables on Salmonellosis Incidence in Kermanshah, West of Iran: A Generalized Linear Model With Negative Binomial Approach. J Environ Health Sci Eng (2021) 19(1):1171–7. 10.1007/s40201-021-00684-z 34150303 PMC8172766

[53] Onozuka D Hashizume M . Weather Variability and Paediatric Infectious Gastroenteritis. Epidemiol and Infect (2011) 139(9):1369–78. 10.1017/S0950268810002451 21044404

[54] Wang H Di B Zhang T Lu Y Chen C Wang D Association of Meteorological Factors with Infectious Diarrhea Incidence in Guangzhou, Southern China: A Time-Series Study (2006–2017). Sci total Environ (2019) 672:7–15. 10.1016/j.scitotenv.2019.03.330 30954825

[55] Baek K Choi J Park J-T Kwak K . Influence of Temperature and Precipitation on the Incidence of Hepatitis A in Seoul, Republic of Korea: A Time Series Analysis Using Distributed Lag Linear and Nonlinear Model. Int J Biometeorology (2022) 66(9):1725–36. 10.1007/s00484-022-02313-2 35829753

[56] Hines JZ Jagger MA Jeanne TL West N Winquist A Robinson BF Heavy Precipitation as a Risk Factor for Shigellosis Among Homeless Persons during an outbreak—Oregon, 2015–2016. J Infect (2018) 76(3):280–5. 10.1016/j.jinf.2017.11.010 29217465 PMC8596496

[57] Stephen DM Barnett AG . Effect of Temperature and Precipitation on Salmonellosis Cases in South‒East Queensland, Australia: An Observational Study. BMJ open (2016) 6(2):e010204. 10.1136/bmjopen-2015-010204 PMC4769393 26916693

[58] Deshpande A Chang HH Levy K . Heavy Rainfall Events and Diarrheal Diseases: The Role of Urban–Rural Geography. The Am J Trop Med Hyg (2020) 103(3):1043–9. 10.4269/ajtmh.19-0768 32700663 PMC7470540

[59] Zhang F Liu Z Gao L Zhang C Jiang B . Short-term Impacts of Floods on Enteric Infectious Disease in Qingdao, China, 2005–2011. Epidemiol and Infect (2016) 144(15):3278–87. 10.1017/S0950268816001084 27312685 PMC9150198

[60] Liu X Liu Z Zhang Y Jiang B . The Effects of Floods on the Incidence of Bacillary Dysentery in Baise (Guangxi Province, China) from 2004 to 2012. Int J Environ Res Public Health (2017) 14(2):179. 10.3390/ijerph14020179 28208681 PMC5334733

[61] Liu Z Ding G Zhang Y Lao J Liu Y Zhang J Identifying Different Types of Flood–Sensitive Diarrheal Diseases from 2006 to 2010 in Guangxi, China. Environ Res (2019) 170:359–65. 10.1016/j.envres.2018.12.067 30623882

[62] Ni W Ding G Li Y Li H Liu Q Jiang B . Effects of the Floods on Dysentery in North Central Region of Henan Province, China from 2004 to 2009. J Infect (2014) 69(5):430–9. 10.1016/j.jinf.2014.05.016 24955987

[63] Zhang N Song D Zhang J Liao W Miao K Zhong S The Impact of the 2016 Flood Event in Anhui Province, China on Infectious Diarrhea Disease: An Interrupted Time-Series Study. Environ Int (2019) 127:801–9. 10.1016/j.envint.2019.03.063 31051323

[64] Xu X Ding G Zhang Y Liu Z Liu Q Jiang B . Quantifying the Impact of Floods on Bacillary Dysentery in Dalian City, China, from 2004 to 2010. Disaster Med Public Health preparedness (2017) 11(2):190–5. 10.1017/dmp.2016.90 27229186

[65] Ni W Ding G Li Y Li H Jiang B . Impacts of Floods on Dysentery in Xinxiang City, China, During 2004–2010: A Time-Series Poisson Analysis. Glob Health Action (2014) 7(1):23904. 10.3402/gha.v7.23904 25098726 PMC4124174

[66] Milojevic A Armstrong B Hashizume M McAllister K Faruque A Yunus M Health Effects of Flooding in Rural Bangladesh. Epidemiology (2012) 23(1):107–15. 10.1097/EDE.0b013e31823ac606 22082995

[67] Ma Y Wen T Xing D Zhang Y . Associations Between Floods and Bacillary Dysentery Cases in Main Urban Areas of Chongqing, China, 2005–2016: A Retrospective Study. Environ Health Prev Med (2021) 26:49–9. 10.1186/s12199-021-00971-z 33874880 PMC8056597

[68] Liao W Wu J Yang L Benmarhnia T Liang X-Z Murtugudde R Detecting the Net Effect of Flooding on Infectious Diarrheal Disease in Anhui Province, China: A Quasi-Experimental Study. Environ Res Lett (2020) 15(12):125015. 10.1088/1748-9326/abccf5

[69] Carlton EJ Woster AP DeWitt P Goldstein RS Levy K . A Systematic Review and Meta-Analysis of Ambient Temperature and Diarrhoeal Diseases. Int J Epidemiol (2016) 45(1):117–30. 10.1093/ije/dyv296 26567313 PMC4881833

[70] Kraay AN Man O Levy MC Levy K Ionides E Eisenberg JN . Understanding the Impact of Rainfall on Diarrhea: Testing the Concentration-Dilution Hypothesis Using a Systematic Review and Meta-Analysis. Environ Health Perspect (2020) 128(12):126001. 10.1289/EHP6181 33284047 PMC7720804

[71] Xin X Jia J Hu X Han Y Liang J Jiang F . Association Between Floods and the Risk of Dysentery in China: A Meta-Analysis. Int J Biometeorology (2021) 65:1245–53. 10.1007/s00484-021-02096-y 33660029

[72] Yazdi MS Ardalan MA Hosseini M Hami Z Heidari R Mosaed R Infectious Diarrhea Risks as a Public Health Emergency in Floods; a Systematic Review and Meta-Analysis. Arch Acad Emerg Med (2024) 12(1):e46–e. 10.22037/aaem.v12i1.2284 38962364 PMC11221827

