# Supplementary material for: Impact of Climate Variability on Foodborne Diarrheal Disease: Systematic Review and Meta-Analysis
Source: Public Health Rev. 2025 Feb 19;46:1607859. doi: 10.3389/phrs.2025.1607859 (PMC11879746; doi:10.3389/phrs.2025.1607859)
Supplement: Supplementary file 3 [file DataSheet6.docx]

**Supplementary File 6**

Association between relative humidity and food-borne diarrheal disease based on the age group of the study participants (Figure 1)

Figure 1: Association between relative humidity and food-borne diarrheal disease based on the age group of the study participants, 2024
