# Supplementary material for: Impact of Climate Variability on Foodborne Diarrheal Disease: Systematic Review and Meta-Analysis
Source: Public Health Rev. 2025 Feb 19;46:1607859. doi: 10.3389/phrs.2025.1607859 (PMC11879746; doi:10.3389/phrs.2025.1607859)
Supplement: Supplementary file 4 [file DataSheet3.DOCX]

**Supplementary File 3**

Study selection process for the included articles (Figure 1)

 **Figure 1:** Study selection process for the included articles to determine the impacts of Climatic variability on foodborne disease and diarrheal disease.
