# Supplementary material for: Impact of Climate Variability on Foodborne Diarrheal Disease: Systematic Review and Meta-Analysis
Source: Public Health Rev. 2025 Feb 19;46:1607859. doi: 10.3389/phrs.2025.1607859 (PMC11879746; doi:10.3389/phrs.2025.1607859)
Supplement: Supplementary file 5 [file DataSheet9.DOCX]

**Supplementary File 9**

Association between rainfall and food-borne diarrheal disease based on the age group of the study participants (Figure 1).

Wangdi and Clements, 2017

Deshpande et al., 2020

Aik et al., 2018

Luque Fernández et al., 2009

Li et al., 2015

Phung et al., 2015

Bhandari et al., 2020

Alemayehu et al., 2020

Singh et al., 2021

Dharmayanti et al., 2022

Wang et al., 2018

Wang et al., 2018

Azage et al., 2017

**All age**

**Children**

**Overall**

Heterogeneity: I^2^

= 89.77%

Heterogeneity: I^2^

= 99.71%

Heterogeneity I^2^

= 0.00, I

2

= 99.96%, H

2

= 2846.83

Test of group differences: Q_b_

(1) = 5.62, p = 0.02

Authors

1

1.2

1.4

1.6

with 95% CI

Effect size

1.05 [

1.35 [

1.01 [

1.02 [

1.00 [

1.05 [

1.01 [

1.00 [

1.00 [

1.00 [

1.00 [

1.02 [

1.00 [

1.03 [

1.00 [

1.01 [

1.05,

1.12,

1.00,

1.01,

1.00,

1.04,

1.00,

1.00,

1.00,

1.00,

1.00,

1.01,

1.00,

1.01,

1.00,

1.00,

1.05]

1.58]

1.01]

1.04]

1.00]

1.07]

1.01]

1.00]

1.00]

1.00]

1.01]

1.03]

1.00]

1.05]

1.01]

1.02]

8.63

0.20

8.35

7.30

8.63

7.30

8.43

8.63

8.63

8.63

8.57

8.06

8.63

(%)

Weight

Random-effects model

Figure 1: Association between rainfall and food-borne diarrheal disease based on the age group of the study participants, 2024
