# Supplementary material for: Impact of Climate Variability on Foodborne Diarrheal Disease: Systematic Review and Meta-Analysis
Source: Public Health Rev. 2025 Feb 19;46:1607859. doi: 10.3389/phrs.2025.1607859 (PMC11879746; doi:10.3389/phrs.2025.1607859)
Supplement: Supplementary file 7 [file DataSheet10.DOCX]

**Supplementary File 10**

Pooled incidence of foodborne disease associated with rainfall after extreme value was removed (Figure 1)

Figure 1: Pooled incidence of foodborne disease associated with rainfall after extreme value was removed, 2024.
