# Supplementary material for: Impact of Climate Variability on Foodborne Diarrheal Disease: Systematic Review and Meta-Analysis
Source: Public Health Rev. 2025 Feb 19;46:1607859. doi: 10.3389/phrs.2025.1607859 (PMC11879746; doi:10.3389/phrs.2025.1607859)
Supplement: Supplementary file 8 [file DataSheet2.DOCX]

**Supplementary File 2**

The JBI critical appraisal tools with nine evaluation criteria; (1) appropriate sampling frame; (2) proper sampling technique; (3) adequate sample size; (4) description of the study subject and setting description; (5) sufficient data analysis; (6) use of valid methods for the identified conditions; (7) valid measurement for all participants; (8)use of appropriate statistical analysis; and (9) adequate response rate.
