# Supplementary material for: Impact of Climate Variability on Foodborne Diarrheal Disease: Systematic Review and Meta-Analysis
Source: Public Health Rev. 2025 Feb 19;46:1607859. doi: 10.3389/phrs.2025.1607859 (PMC11879746; doi:10.3389/phrs.2025.1607859)
Supplement: Supplementary file 10 [file DataSheet1.DOCX]

**Supplementary File 1**

**Search strategy for PubMed**

The main key terms, such as climate, climate change, climatic factors, meteorological factors, temperature, humidity, rainfall, precipitation, health, extreme events, food borne disease, diarrhea, and foodborne pathogen will be used to retrieve the articles from the included sources. The Boolean operator (AND, OR and NOT) will be used to combine search terms across the include electronic databases or sources.

At least the following search will made to retrieve the articles from PubMed:-

1. ((("climate change"[MeSH Terms] OR ("climate"[All Fields] AND "change"[All Fields]) OR "climate change"[All Fields]) AND "change"[All Fields]) OR Variability[All Fields]) AND ("foodborne diseases"[MeSH Terms] OR ("foodborne"[All Fields] AND "diseases"[All Fields]) OR "foodborne diseases"[All Fields] OR ("foodborne"[All Fields] AND "disease"[All Fields]) OR "foodborne disease"[All Fields])
2. (climate change[Title] OR "climate change"[MeSH Terms]) OR climate variability[Title]) OR precipitation[Title]) OR temperature[Title]) OR "temperature"[MeSH Terms]) OR humidity[Title]) OR "humidity"[MeSH Terms]) OR rainfall[Title]) OR heat[Title]) OR "hot temperature"[MeSH Terms]) AND foodborne disease[Title]) OR "foodborne diseases"[MeSH Terms]) OR salmonella[Title]) OR "salmonella"[MeSH Terms]) OR diarrhoea[Title]) OR "diarrhea"[MeSH Terms]) OR campylobacter[Title]) OR "campylobacter"[MeSH Terms]) OR listeria monocytogenes[Title]) OR "listeria monocytogenes"[MeSH Terms].

The same key terms were used for other databases to retrieve articles from other databases, with the modification.
